# Application of serum SERS technology combined with deep learning algorithm in the rapid diagnosis of immune diseases and chronic kidney disease

**DOI:** 10.1038/s41598-023-42719-5

**Published:** 2023-09-21

**Authors:** Jie Yang, Xiaomei Chen, Cainan Luo, Zhengfang Li, Chen Chen, Shibin Han, Xiaoyi Lv, Lijun Wu, Cheng Chen

**Affiliations:** 1 College of Information Science and Engineering, Xinjiang University, Urumqi, 830046 China; 2 Department of Rheumatology and Immunology, People’s Hospital of Xinjiang Uygur Autonomous Region, Urumqi, 830001 China; 3 Xinjiang Clinical Research Center for Rheumatoid arthritis, Urumqi, 830001 China; 4 Xinjiang Medical University, Urumqi, 830054 China; 5 College of Physics Science and Technology, Xinjiang University, Urumqi, 830046 China; 6 College of Software, Xinjiang University, Urumqi, 830046 China

**Keywords:** Raman spectroscopy, Machine learning

## Abstract

Surface-enhanced Raman spectroscopy (SERS), as a rapid, non-invasive and reliable spectroscopic detection technique, has promising applications in disease screening and diagnosis. In this paper, an annealed silver nanoparticles/porous silicon Bragg reflector (AgNPs/PSB) composite SERS substrate with high sensitivity and strong stability was prepared by immersion plating and heat treatment using porous silicon Bragg reflector (PSB) as the substrate. The substrate combines the five deep learning algorithms of the improved AlexNet, ResNet, SqueezeNet, temporal convolutional network (TCN) and multiscale fusion convolutional neural network (MCNN). We constructed rapid screening models for patients with primary Sjögren’s syndrome (pSS) and healthy controls (HC), diabetic nephropathy patients (DN) and healthy controls (HC), respectively. The results showed that the annealed AgNPs/PSB composite SERS substrates performed well in diagnosing. Among them, the MCNN model had the best classification effect in the two groups of experiments, with an accuracy rate of 94.7% and 92.0%, respectively. Previous studies have indicated that the AgNPs/PSB composite SERS substrate, combined with machine learning algorithms, has achieved promising classification results in disease diagnosis. This study shows that SERS technology based on annealed AgNPs/PSB composite substrate combined with deep learning algorithm has a greater developmental prospect and research value in the early identification and screening of immune diseases and chronic kidney disease, providing reference ideas for non-invasive and rapid clinical medical diagnosis of patients.

## Introduction

Primary Sjogren’s syndrome (pSS) and diabetic nephropathy (DN) are a chronic disease^1,2^. As an autoimmune disease, the average prevalence of pSS is 0.06%, among them, women are 9 times more than men^3^. An estimated 35 million people worldwide will be affected^4,5^. DN is one of the world’s most common chronic kidney diseases, diabetes accounts for 11.3% of global deaths. Some studies suggest that by 2025, 472 million people will have diabetes worldwide^6–8^. In addition, pSS and DN can lead to severe complications^9,10^. As the number of patients continues to grow in recent years, it has caused severe economic pressure on the state, society and individuals. Therefore, early screening is vital to reduce the incidence of pSS and DN and to improve disease management and patient prognosis. Currently, salivary gland biopsy is an essential indicator for the diagnosis of pSS^11^. However, this is an invasive method, and the accurate interpretation of the biopsy is susceptible to the subjective experience of the physician, leading to misdiagnosis. The Glucose Tolerance Test (OGTT) method is the “gold standard” for the early diagnosis of DN^7^. However, the test is complex and time-consuming, and it can cause discomfort and increase the psychological burden of patients, making it unsuitable for mass population screening.

Raman spectroscopy is a non-invasive, fast and accurate optical detection technique. It can give fingerprint information on molecular structure and content^12^ and has raised much interest in medical screening, environmental protection, and food safety^13–15^. However, in practical applications, the weak signal intensity of Raman spectroscopy, small scattering cross section and susceptibility to fluorescence interference limit its application in molecular detection. Surface-enhanced Raman spectroscopy (SERS), a method based on adsorption on metal nanostructures to amplify the Raman signal of analytes, can enhance the signal to 10^10^–10^14^ times in the active matrix, increasing the sensitivity of Raman spectroscopy as much as possible^16,17^. At present, SERS technology has become a promising bioassay technology for a wide range of applications in cancer, genetic diseases and infectious diseases^18–20^. With the rise of SERS technology, there is also increasing interest in choosing SERS metal substrates. Numerous studies have shown that the silver nanoparticles (AgNPs) structure has good stability and reproducibility and is a low-cost and scalable method^21–23^, so AgNPs have become the preferred SERS substrate for researchers. In addition, in studies focusing on SERS detection, related reports indicate that porous silicon (PSi) is a rather suitable SERS substrate^24–26^. It has many favorable properties, such as large surface area, open porous structure and non-toxicity^25,27^. In recent years, people have tried to combine PSi with nano-precious metal particles to apply SERS technology, and outstanding achievements have also been made. For example, in 2020, Wali et al. prepared a new generation of AuNPs/PSi SERS-active substrates with strong enhanced performance and reproducibility, and the substrates exhibited efficient detection capabilities^28^. In 2021, Gao et al. designed a SERS substrate to diagnose cervical and breast cancer. Combining gold nanoparticles with porous silicon photonic crystals with a central wavelength of 785 nm has prepared a SERS substrate with excellent performance. It has realized cervical and breast cancer detection in clinical medicine^16^. In addition, annealing helps to optimize the particle size and morphological structure of silver nanoparticles, which further optimizes the electric field distribution of silver nanoparticles, thus enabling more sensitive SERS performance^29,30^.

SERS technology is widely used in the biomedical field because it can significantly enhance the Raman signal of biological samples such as serum and urine. Its combination with deep learning algorithms can further expand the application of SERS technology to achieve rapid screening of diseases. Related studies have shown that deep learning algorithms better analyze spectral signals, including Raman spectra^31–33^. Recently, Raman spectroscopy combined with deep learning algorithms has been well used in disease detection and diagnosis. For example, Chen et al. used various deep learning algorithms, such as multilayer perceptron (MLP) and recursive neural network (RNN), combined with serum Raman spectroscopy, achieved screening and diagnosis of glioma, and achieved better results^34^. At the same time, in diagnosing other diseases, there are also many applications of deep learning combined with SERS technology. For example, Cheng et al. fabricated a SERS substrate consisting of a composite of Au–Ag nanocomplexes and ZnO nanopillars. They combined it with a CNN classifier and achieved an innovative biosensing method for liver disease^35^. Shin et al. collected plasma samples from healthy controls and lung cancer patients and obtained SERS spectra of exosomes. They used deep learning spectral analysis of SERS technology to identify lung cancer patients. Finally, the feasibility of combining SERS spectroscopy with deep learning in screening plasma samples for lung cancer was shown^33^. In general, the combination of SERS technology and deep learning algorithm has made a tremendous breakthrough in clinical medical diagnosis and is full of great potential in the future^35^.

In this study, we fabricated an annealed AgNPs/PSB composite SERS substrate with high sensitivity and strong stability by immersion plating and heat treatment using PSB as the substrate. In addition, reproducible and simple silicon-based SERS substrate preparation processes and good biocompatibility offer opportunities for the commercial development of SERS substrates. In previous studies, the stability of the AgNPs/PSB composite SERS substrate has also been demonstrated. Feature extraction was conducted using PCA, and in combination with machine learning, an SVM classification model was established, leading to favorable diagnostic outcomes^36^. In this paper, to further verify the potential of the SERS substrate and technology in disease diagnosis, we constructed two groups of experiments for HC/pSS and HC/DN. The experiment established five classification models based on serum SERS spectrum combined with deep learning algorithm: AlexNet, ResNet, SqueezeNet, TCN and MCNN. Both sets of experimental results showed that the combination of SERS spectroscopy and deep learning algorithms could quickly and effectively distinguish healthy controls from chronic disease patients. Therefore, this study shows the feasibility of the SERS technology based on the annealed AgNPs/PSB composite substrate combined with deep learning algorithms for the diagnosis of immune diseases and chronic kidney diseases. From the perspective of SERS spectrum, it provides an interesting and effective reference idea for the rapid identification and screening of immune diseases and chronic kidney diseases.

## Materials and methods

### Chemicals

The silicon wafers were purchased from Tianjin Semiconductor Research Institute. Silver nitrate (AgNO_3_), hydrofluoric acid (HF) and ethanol (CH_3_CH_2_OH) were purchased from Sinopharm Chemical Reagent Co. All were analytical standards available for use with no purification of any kind. The entire experiment used ionized water.

### Experimental materials

All samples in our study were obtained from the People’s Hospital of Xinjiang Uygur Autonomous Region and were approved by the Ethics Committee of the People's Hospital of Xinjiang Uygur Autonomous Region (KY20220311003). All studies were conducted in accordance with relevant guidelines/regulations, and informed consent was obtained from all participants. In this experiment, a total of 9 pSS patients, 10 DN patients and 7 healthy controls were collected, and fresh blood samples were collected. Table 1 contains demographic information about the patients and healthy controls, such as their age and sex. Anticoagulant-free peripheral blood was drawn. Centrifuge at 4000 r/min for 5 min at 4 °C, and then obtained the serum from the uppermost clear night, and stored it in a − 80 °C refrigerator for subsequent spectrum collection.

**Table 1:** Demographic information of pSS patients, DN patients and healthy controls.

	pSS	DN	HC
	(n = 9)	(n = 10)	(n = 7)
Age			
Mean	51.2(± 7.9)	53.6(± 8.1)	52.5(± 8.3)
Gender			
Male	3	6	2
Female	6	4	5

### Preparation of SERS substrates

In our method, PSB was first prepared by anodic electrochemical etching. A p-type boron-doped single crystal silicon wafer (crystal orientation < 100 >) with the resistivity of 0.03–0.06 Ω·cm was cut into 2 × 2 cm^2^ squares. Then acetone, anhydrous ethanol and deionized water in the ultrasonic cleaner for 10 min to reduce the impact of impurities such as dust and grease on corrosion. Then put the silicon wafer into the etching tank, among them, wherein the etching tank was made of polytetrafluoroethylene, and the etching solution in the etching tank was composed of HF and anhydrous ethanol according to the mixed concentration ratio of 1:1. Then the Labview program was used to set the high and low refractive index layers, where the current densities were 65 mA/cm^2^ and 115 mA/cm^2^, and the etching times were 1.2 s and 1 s, respectively. In the corrosion process, to ensure sufficient fluoride and the uniformity of corrosion, each layer of PSi was formed in a ventilated environment with a time interval of 3 s. The corroded PSi was then rinsed with ionized water and dried in a nitrogen atmosphere^36^.

Then AgNPs were reduced on the PSB surface by taking advantage of a large number of Si–H bonds on the PSB and the reducing nature of the bonds. In the experiment, we immersed the prepared PSB substrate in AgNO_3_ solution with a concentration of 0.01 M and used the immersion plating method to reduce AgNPs on the PSB in situ.

Finally, annealing treatment was performed, and the prepared substrate was annealed using a muffle furnace. We annealed at 300 °C for 1 h to complete the annealing of the AgNPs/PSB substrate.

### SERS data acquisition

**Figure 1:**
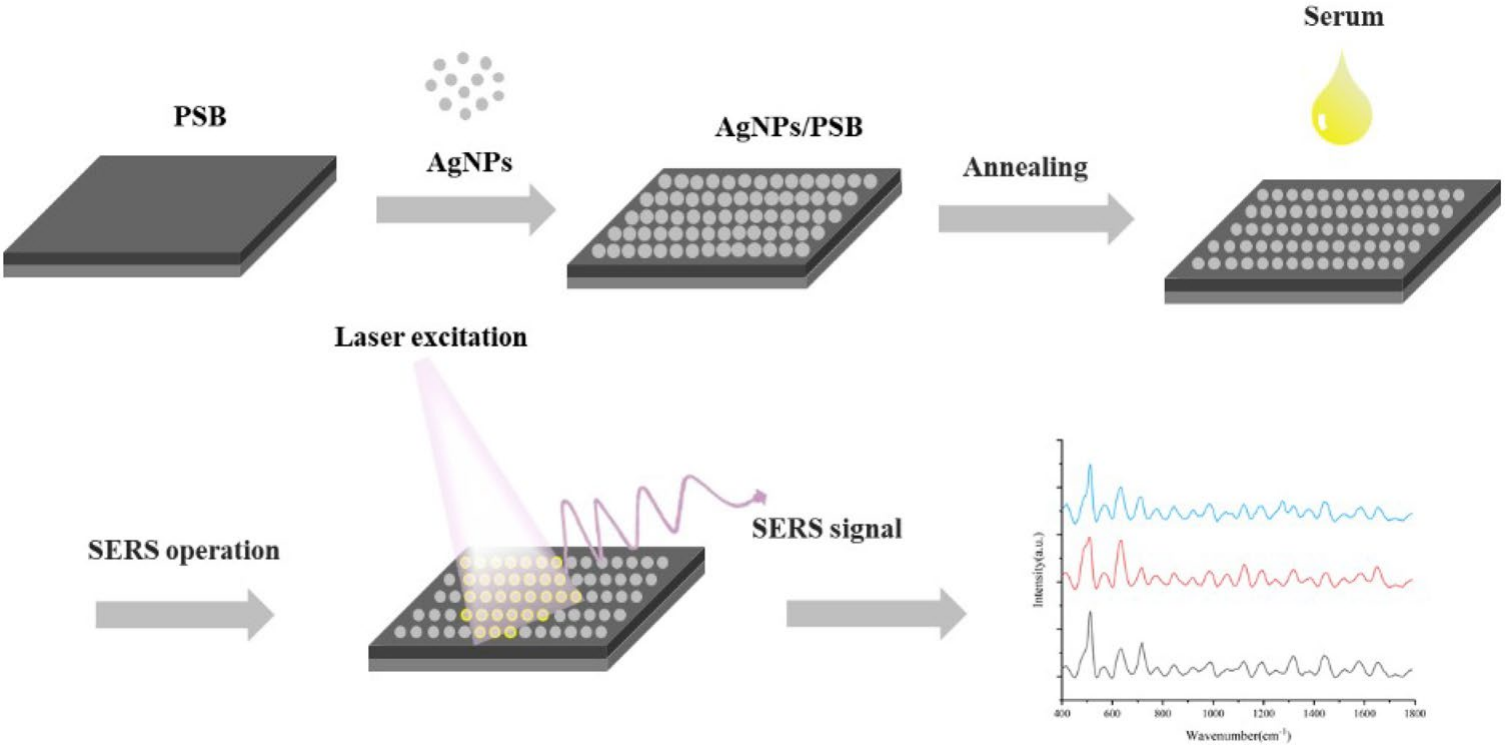
Serum SERS measurement protocol.

The SERS measurement scheme of serum samples on AgNPs/PSB substrates is shown in Fig. 1. SERS spectra of serum samples were acquired using a high-resolution confocal Raman spectrometer (LabRAM HR Evolution, gora Raman spectroscopy, ideaoptics, China). The laser wavelength was 785 nm, the laser power was 160 mW, the power irradiated on the sample surface was 112 mW, the objective lens specification (NA = 10 × , the laser spot size was 2.2 μm), and the spectral resolution of the spectrometer was 5 cm^–1^, and the integration time was 15 s. The laser beam was focused on the sample surface by a 10 × mirror, with a spectral range from 400 to 1800 cm^−1^. Different positions of each sample were measured 3 times, and 27 spectra were obtained for pSS patients, 30 spectra for DN patients and 21 spectra for HC, for a total of 78 spectral data.

### Data preprocessing

Since the serum SERS spectrum collected by the spectrometer is interfered with by factors such as measurement conditions, detection environment and hardware facilities, the spectrum is complex, which will affect the analysis effect to a large extent. Therefore, performing a preprocessing operation on the collected spectral data is necessary. In the experiment, we corrected by using the morpho3.2 software in the instrument (Version 3.2.26, Shanghai Fuxiang Optics Co., Ltd, Building 4, No. 200 Guoding East Road, Yangpu District, Shanghai). The instrument response in the spectrum was corrected by subtracting the dark current, detector response and optical system signal. The adaptive iteratively reweighted penalized least squares (airPLS) method has the advantages of simplicity, convenience and flexibility^37,38^, and is the most widely used method for baseline correction in recent years. It mainly introduces sparse matrix technology and adaptive iterative technology, which can quickly fit the difference between the baseline and the original signal^39^. Therefore, in this paper, we used the airPLS method for baseline correction. The airPLS parameters were set as follows: the number of iterations was 500, the threshold was 0.1, the asymmetry factor was 0.05, and the number of baseline points was 30. Then the SERS spectrum was linearly normalized to convert the input spectral data to the range of [0–1]. Its formula is:

1 $$ y = \frac{{x_{1} - x_{\min } }}{{x_{\max } - x_{\min } }}, $$

Among them, ${x}_{l}$ and $y$ are the values before and after normalization, respectively, and ${x}_{max}$ and $x$_min_ are the maximum and minimum values of the sample data, respectively. This can eliminate noise interference and reduce data complexity, thus improving the convergence speed^40^. The preprocessing of this experiment was carried out in Matlab2016b.

### Classification model

**Figure 2:**
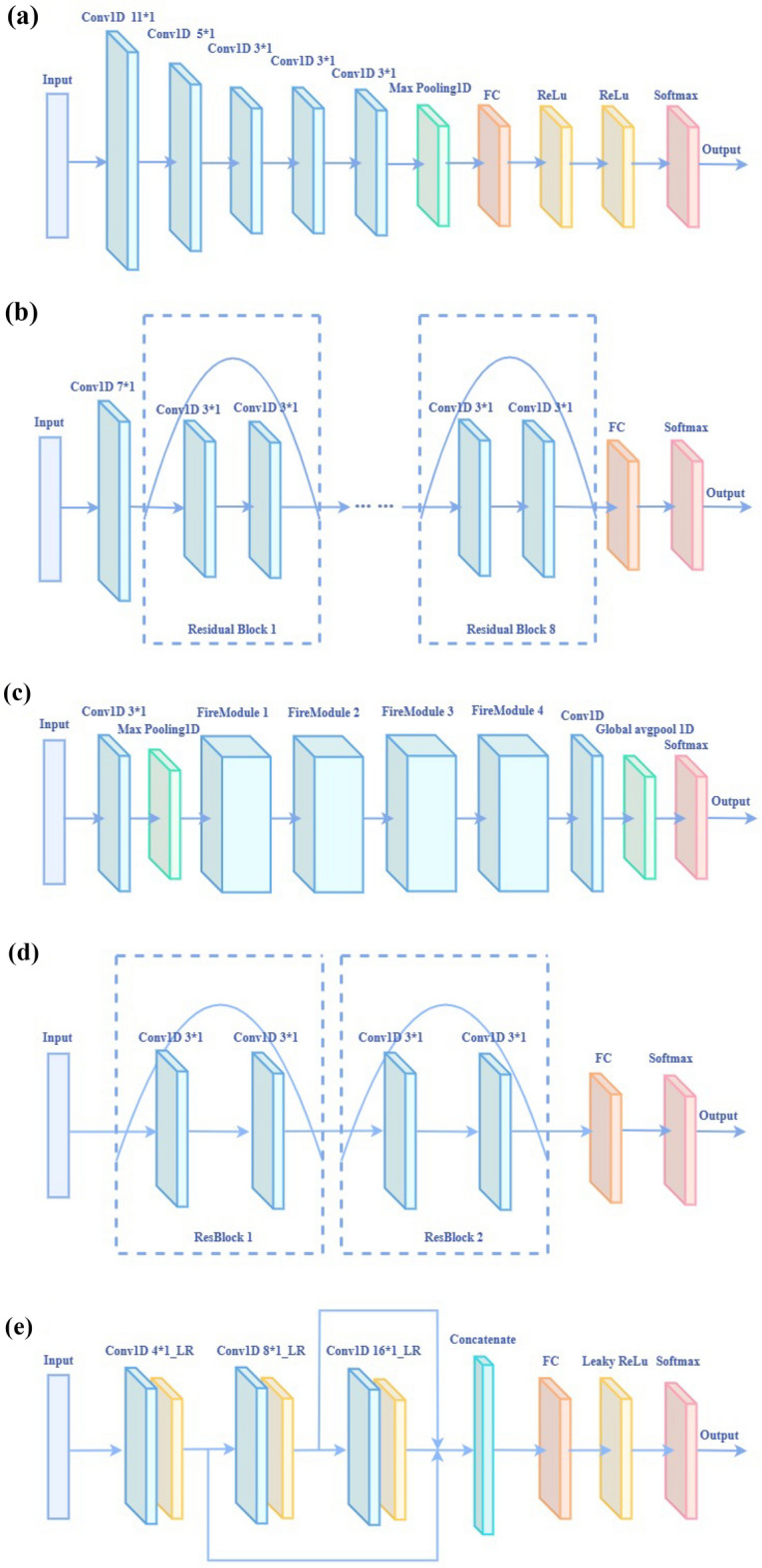
The structure diagram of the five models after fine-tuning: (**a**) AlexNet model, (**b**) ResNet model, (**c**) SqueezeNet model, (**d**) TCN model, (**e**) MCNN model.

Deep learning has become a hot topic of discussion in artificial intelligence, capable of efficiently processing data such as images, speech, and texts^41^. With the vigorous growth of the deep learning industry and the update of computer hardware, deep learning has shown its advantages and capabilities over traditional machine learning. It is now commonly used in medical diagnostics, food safety and bioinformatics. In addition, improvements to the model will further improve deep learning performance. Deep learning also has a higher fault tolerance and greater adaptability compared to machine learning^42^. Similarly, deep learning has recently been applied to spectral signal analysis^32^. Therefore, in the future, deep learning will have more excellent development prospects and application value in research. In this study, the models we constructed are AlexNet, ResNet, SqueezeNet, TCN and MCNN. Among them, the first four are deep learning algorithms with broad influence in recent years, and MCNN is a novel model we have designed. During the experiment, all the 2D convolutional layers of the model were changed to 1D convolutional layers. The selected optimizer was Adam. The number of iterations was 200, the batch was set to 24, and the learning rate was 0.000001. Figure 2 shows the structure of the five models. The classification model was established and realized through Python3.7.6. The code for this study was available at https://github.com/tianqiong619/The-five-neural-networks.

AlexNet is the classical CNN model, and its appearance significantly advanced the progress of deep learning. AlexNet can not only effectively avoid gradient dispersion but also has significant advantages for handling complex data^43^. Due to this study's small number of samples, the 1-dimensional spectral data contained a relatively small number of features. To improve the performance, we made improvements to AlexNet. The structure of the fine-tuned AlexNet model is shown in Fig. 2a. The model consists of five 1D convolutional layers, the aggregation layers of the first two convolutional layers are deleted, and the Relu activation function is used, with a Dropout of 0.5 and 0.2 for the fully connected layers to prevent overfitting of the model. The input data is feature extracted through multiple convolutional layers, and then the information extracted by the convolutional layer is compressed through the pooling layer. The flatten layer converts the data output by the pooling layer into one-dimensional data, and then passes through two ReLu activation layers, and finally passes through the softmax function outputs classification probabilities.

ResNet innovatively introduces the residual module on the neural network. The residual block adopts the idea of constant mapping, and the use of shortcut connections effectively solves the problems of vanishing and exploding gradients, as well as model degradation in deep networks. Compared with the regular model, a short-circuit mechanism is added to enable deeper layers to function through residual learning^44^. Figure 2b shows this study’s structure of the ResNet network. The model contains eight residual blocks, where the activation functions of the convolution and dense layers use ReLu and softmax, respectively. The convolutional layer performs feature extraction on the input data, and then passes through eight residual blocks in turn, and then the flatten layer converts the output data of the residual block into one-dimensional data, and finally outputs the classification result of the spectral data through softmax.

SqueezeNet is a classic lightweight network that maximizes computing efficiency without reducing model accuracy, not only optimizing the network but also lowering the computational cost^45^. SqueezeNet takes a different approach from traditional convolution and proposes the Fire module. Among them, the Fire module is composed of 3 convolutional layers, including 1 squeeze module and 2 extension modules. Figure 2c shows this study’s structure of the SqueezeNet network. The SqueezeNet model includes four Fire modules. The spectral data first passes through the convolutional layer and the pooling layer to extract and compress the local information of the original features, then passes through the four Fire modules in turn, and then passes through the convolutional layer with a kernel of 2, and finally the results of model processing are output through the global avgpool layer and softmax layer.

TCN is an advanced time series processing network. The expanded TCN architecture consists of residual blocks containing causal convolutional layers. Causal convolution continues the way CNN imitates LSTM, which other CNN models do not have^46^. Because the object of study is spectral data, the designed TCN uses a one-dimensional convolutional network. The fine-tuned TCN model structure is shown in Fig. 2d. The TCN designed in this paper contains two residual blocks, each consisting of two causal convolutional layers. The input data first passes through two residual blocks, then the flatten layer converts the features output by the residual block into one-dimensional data, and finally the softmax function outputs the classification result.

The MCNN model mainly performs feature extraction through different convolutional layers and integrates some novel ideas. The structure of MCNN is shown in Fig. 2e. It mainly consists of three 1D convolutional layers, one flatten layer, and two fully connected layers. In this study, LeakyRelu is used as the activation function, a batch normalization layer (BN) is introduced to improve the model training and convergence speed further, and a dropout layer is added to avoid model overfitting and gradient disappearance. To extract more detailed and sufficient features of spectral samples, we build three convolutional layers, and the kernel size increases with the deepening of the convolutional layers. The input spectral data passes through three convolutional layers and the LeakyRelu layer in turn, then the network fuses the features extracted from the three layers so that features of different depths can be taken into account. The flatten layer then converts the features in the convolutional layer into one-dimensional data and connects a fully connected layer. Finally, softmax outputs the classification results of the SERS spectral data.

### Model metrics

We used different metrics to evaluate the performance of the five classification models, namely Sensitivity, Specificity, Precision, and Accuracy. According to Table 2, the index values were calculated with the formulas as (2, 3, 4 and 5):

2 $$ Sensitivity = \frac{TP}{{TP + FN}}, $$

3 $$ Specificity = \frac{TN}{{FP + TN}}, $$

4 $$ \Pr {\text{e}}cision = \frac{TP}{{TP + FP}}, $$

5 $$ Accuracy = \frac{TP + TN}{{TP + TN + FP + FN}}. $$

**Table 2:** Confusion matrix.

Predicted	Observed	
	Positive	Negative
Positive	TP	FP
Negative	FN	TN

## Results

### SERS effect of serum samples

**Figure 3:**
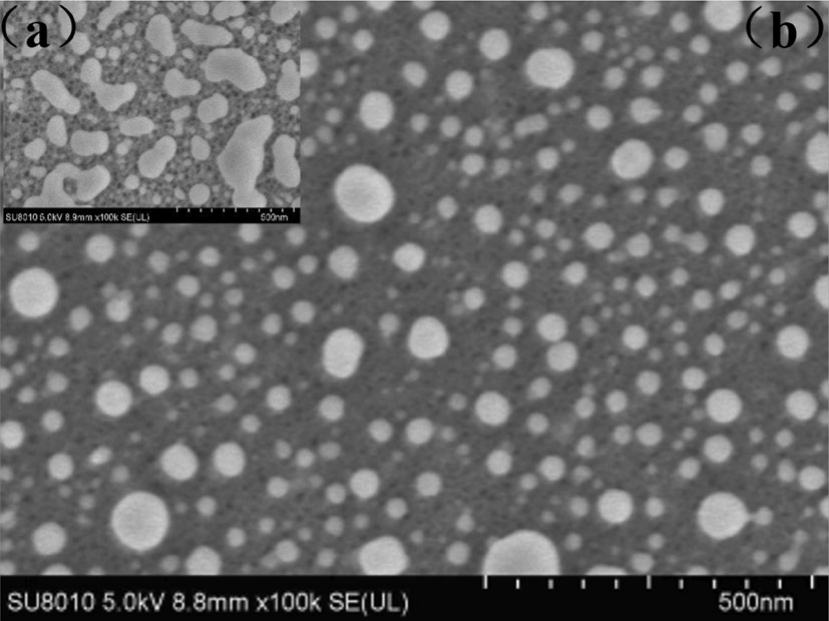
(**a**) and (**b**) Comparison of substrate surface morphology before and after annealing.

**Figure 4:**
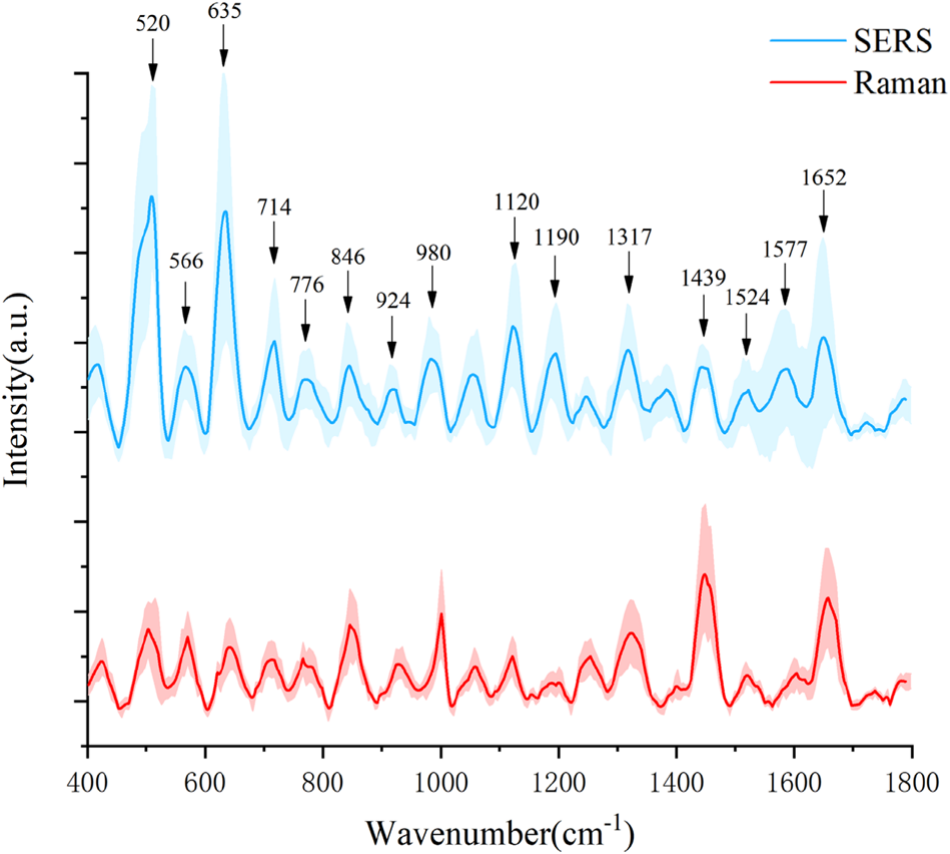
Comparison of SERS spectra and conventional Raman spectra of pSS patients (the shaded area represents the standard deviation of the mean).

Figure 3a and b show the comparison of the surface morphology of the SERS substrate before and after high temperature annealing. The particle size of silver nanoparticles prepared by immersion-plating method before annealing is 80 ~ 200 nm, and the particle size of silver nanoparticles changes obviously after annealing, and the particle size is 20 ~ 70 nm. We compared with the AgNPs before high-temperature annealing, Fig. 3b can show that the particle size of our AgNPs is more uniform after annealing. To demonstrate the enhancement effect of our prepared AgNPs/PSB substrates of sera, we measured serum SERS spectra and conventional Raman spectra of pSS patients on a dry surface. As shown in Fig. 4, we plotted the mean Raman, mean SERS spectra of pSS patient serum samples. Among them, the shaded area represents the standard deviation of the mean. As could be seen from the shaded area in the figure, there were fluctuations in the same spectrum at different peaks. But at the same time, there were significant differences between the two spectra at some peaks, such as at 520 cm^–1^, 635 cm^–1^, 846 cm^–1^, 1120 cm^–1^, 1439 cm^–1^, etc. By comparing the SERS spectrum of the serum sample with the conventional Raman spectrum, it showed that the SERS enhanced data collected in the experiment was helpful to the subsequent classification experiment model.

### Spectral analysis of serum SERS

**Figure 5:**
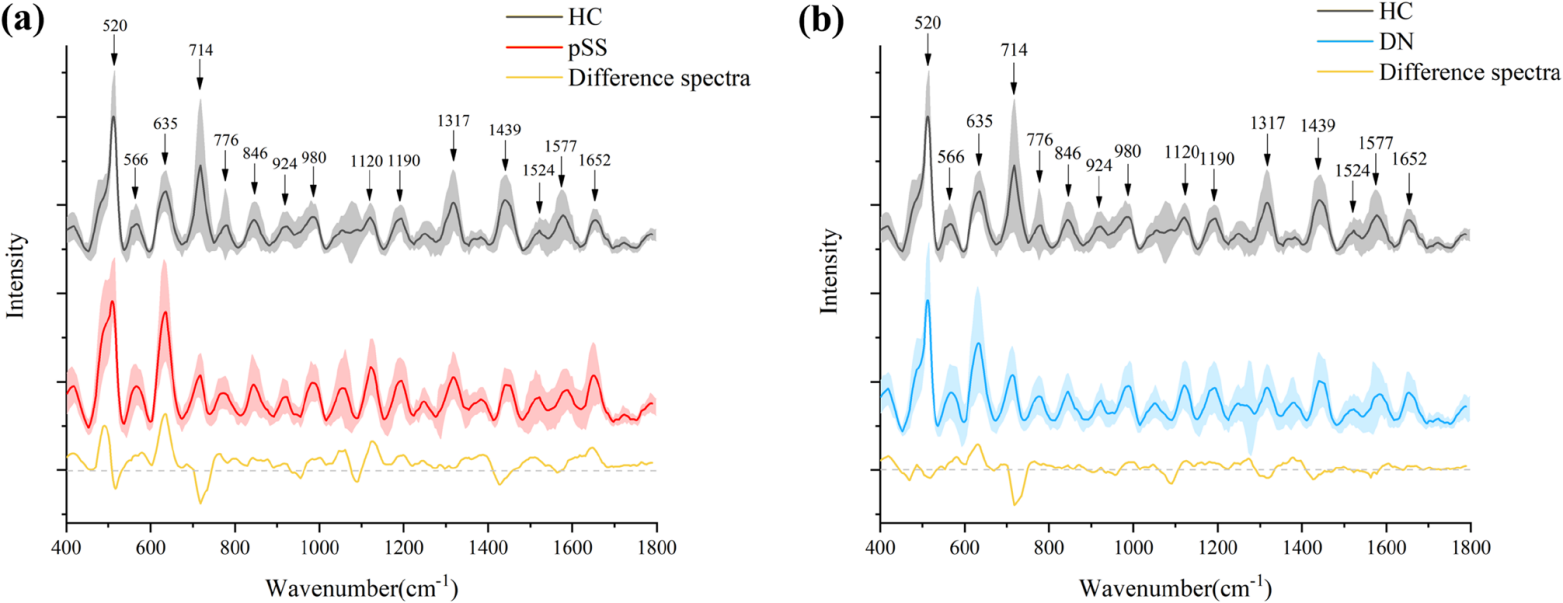
(**a**) is the normalized mean Raman spectrum of HC/pSS (the shaded area represents the standard deviation of the mean). (**b**) is the normalized mean Raman spectrum of HC/DN (the shaded area represents the standard deviation of the mean).

Figure 5 shows the mean serum Raman spectra of HC and pSS, HC and DN after pretreatment over the 400 cm^−1^ to 1800 cm^−1^ range. As shown, the waveforms of the different spectra in patients and healthy controls were similar, but the magnitude of fluctuations differed. The serum SERS spectra of pSS patients peaked at 520, 635, 714, 1120, 1317, 1439 and 1652 cm^−1^, and these peaks also appeared in the SERS spectra of sera from DN patients and HC. The differences in SERS spectra between HC and pSS patients and HC and DN patients demonstrate that the lesions in pSS and DN cause changes in blood composition, resulting in differential SERS spectral intensity between the two groups. This provides an essential theoretical basis for accurately classifying patients and healthy controls.

### Sample division

To prevent the SERS spectra from being confused with the training set and test set data due to random division, and to ensure the real validity of the experimental results. We collected three measurements of each serum sample as a set and divided them into training and test sets in a 7:3 ratio. To measure the model's prediction performance and reduce overfitting, we used a five-fold cross-validation to validate the model and calculate the average of the results of the five runs.

### Data augmentation

Data augmentation is a commonly used method to expand the sample size of the training set, and it can effectively increase the diversity of the training set. In recent years, data augmentation combining spectral data and deep learning has been widely used in disease diagnosis^47^. Among them, data augmentation methods using Gaussian white noise are also used more often in spectroscopy studies^40,48^. To increase the amount of data to improve the generalization ability of the model, in this study, we expanded the training set by a factor of 10 with Gaussian white noise of different intensities. The final spectrum data for pSS was 207, the spectrum data for DN was 240, and the spectrum data for HC was 171.

### Classification model results

**Figure 6:**
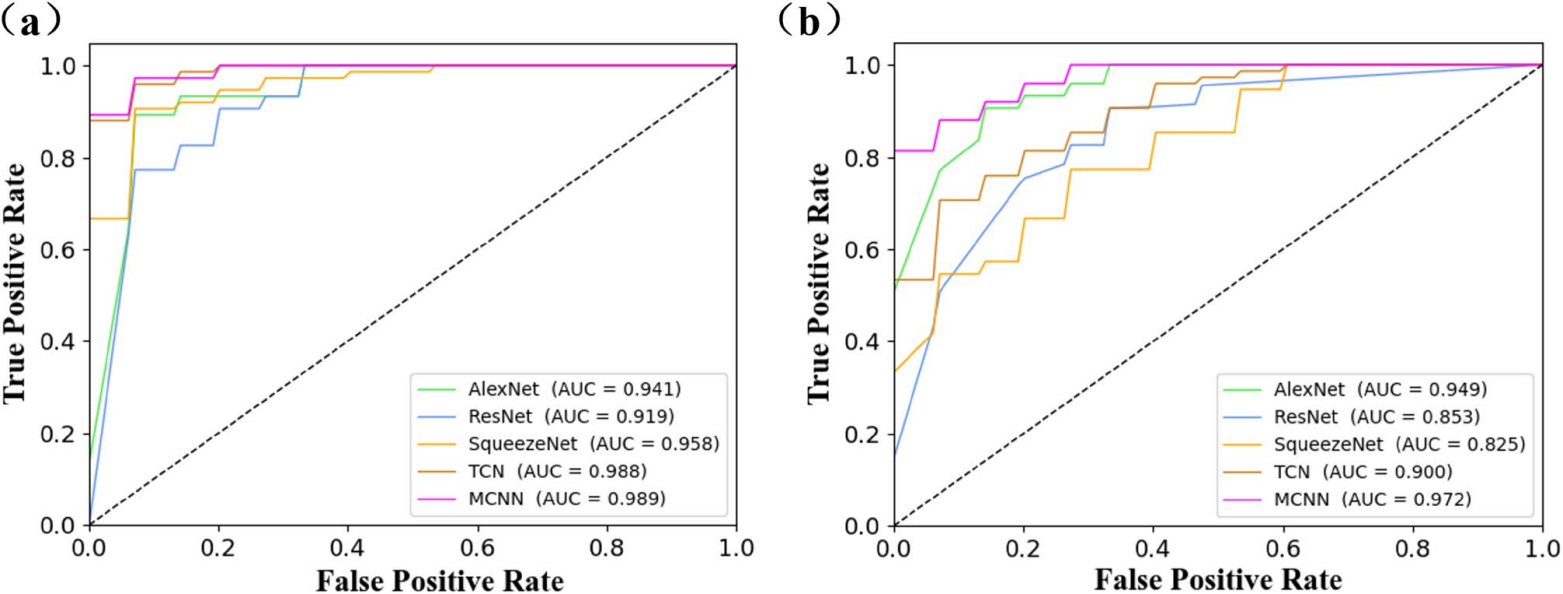
(**a**) ROC curves of five models of HC and pSS. (**b**) ROC curves of five models of HC and DN.

In this experiment, pSS patients, DN patients and HC were divided into two groups. The 1st group was HC and pSS, and the 2nd group was HC and DN. The predicted values and results of the detailed results of the model five-fold cross-validation are shown in Supplementary Tables S1–S20, and the strip plots are shown in Supplementary Material Figs. S1–S50. The results of the five models are shown in Table 3. In the first group of experiments with HC and pSS patients, the MCNN model had the highest classification accuracy of 94.7%. Among them, the accuracy rates of AlexNet, SqueezeNet and TCN models all exceed 85%, while the ResNet model had a lower accuracy rate than other models, which was 82.7%. In order to further evaluate the classification performance of the five models, we plotted the receiver-operating characteristic (ROC) curves. AUC is the area-under-the-ROC curve, and the larger the AUC value, the better the experimental effects. Figure 6 shows the ROC curve of the average results of the model runs. Among them, the AUC of the MCNN model was 0.989, and the AUC of the ResNet model was the lowest, which was 0.919. In the experiments of the second group of HC and DN patients, the MCNN model achieved the best results in terms of accuracy, sensitivity, specificity, precision, and AUC, with 92.0%, 95.6%, 86.7%, 91.6% and 0.972, respectively. The accuracy, sensitivity, specificity, precision and AUC of the SqueezeNet model were not ideal, which were 76.0%, 64.4%, 93.3%, 96.4% and 0.825, respectively. Combining the results of the evaluation indexes of the two experimental models, we believed that the MCNN model had the best discriminatory effect on patients.

**Table 3:** Experimental results of the five models.

Comparison group	Model	Accuracy (%)	Sensitivity (%)	Specificity (%)	Precision (%)	AUC
HC vs pSS	AlexNet	89.3	100	73.3	85.1	0.941
	ResNet	82.7	97.8	60.0	78.7	0.919
	SqueezeNet	92.0	97.8	83.3	89.8	0.958
	TCN	93.3	100	83.3	90.0	0.988
	MCNN	94.7	100	86.7	92.0	0.989
HC vs DN	AlexNet	88.0	95.6	76.7	86.5	0.949
	ResNet	80.0	84.4	73.3	84.0	0.853
	SqueezeNet	76.0	64.4	93.3	96.4	0.825
	TCN	82.7	97.8	60.0	78.9	0.900
	MCNN	92.0	95.6	86.7	91.6	0.972

## Discussion

SERS, as a rapid, non-invasive and reliable spectroscopic detection technique, reflects biomolecules’ rich chemical fingerprint information, such as proteins, nucleic acids and lipids^49,50^. Table 4 lists the major Raman peak positions and assignments of serum SERS spectra. In combination with the spectrograms and Table 4, the Raman peaks located at 566, 635, 776, 846, 924, 980, 1120, 1190 and 1652 cm^−1^ were observed, and the peaks at these bands were higher for pSS and DN patients than for HC. Raman peak at 566 cm^−1^ represents tryptophan, Raman peak at 635 cm^−1^ represents tyrosine, Raman peak at 846 cm^−1^ represents valine, Raman peak at 924 cm^−1^ represents C–C stretching of proline and collagen, Raman peak at 1120 cm^−1^ represents carotene, and Raman peak at 1652 cm^−1^ represents lipid. This suggests that pSS and DN patients have higher levels of tryptophan, tyrosine, valine, carotene and lipid than HC. Raman peaks at 520, 714 and 1439 cm^−1^, the Raman peak intensity of HC was higher than pSS and DN patients. Raman peak at 520 cm^−1^ represents proteins, Raman peak at 714 cm^−1^ represents polysaccharides, and Raman peak at 1439 cm^−1^ represents phospholipids. This suggests that blood composition in patients with pSS and DN has altered, resulting in less concentration of proteins, polysaccharides and phospholipids in patients than in HC. Raman peak at 1317 cm^−1^ represents guanine, Raman peak at 1524 cm^−1^ represents carotenoid, Raman peak at 1577 cm^−1^ represents phenylalanine. The pSS patients at these peaks were higher than HC, while the peaks in DN patients were slightly lower than HC. The difference in Raman spectral intensity at the peak between patients and healthy controls reflects the differences in the content of substances such as polysaccharides, protein and lipids in the human body. Therefore, this provides a feasibility and biological basis for using serum SERS spectroscopy to identify both types of samples.

**Table 4:** Main Raman peak locations and assignments for human serum SERS spectra.

Wavenumber (cm^–1^)	Assignment
520	Proteins^51^
566	Tryptophan^52^
635	Tyrosine^53^
714	polysaccharides^54^
776	Phosphatidylinositol^55^
846	Valine^51^
924	C–C stretching of proline and collagen^56^
980	=CH bending (lipids)^55^
1120	Carotene^57^
1190	Cytosine^58^
1317	Guanine^59^
1439	Phospholipids^54^
1524	Carotenoid^55^
1577	Phenylalanine^60^
1652	Lipid (C=C stretching)^59^

In order to evaluate the accuracy of screening using serum SERS spectrum, we constructed two groups of experiments for HC/pSS and HC/DN, respectively, and proposed a study on the identification of immune diseases and chronic kidney disease using SERS spectrum combined with deep learning algorithms. In order to increase the robustness and generalizability of a model, we introduced data augmentation to the small sample dataset and expanded the training set by a factor of 10 using a Gaussian white noise approach. In addition, to further verify experimental reliability and determine the best classifier, we used a five-fold cross-validation method. The final evaluation criteria were based on the average of five times for each evaluation index. The experimental results show that in the HC and pSS experiments, the MCNN model performs the best, followed by TCN, and ResNet has a lower accuracy compared to other models. In another set of HC and DN experiments, the MCNN model achieved the best results, followed by AlexNet, while the SqueezeNet model had the lowest accuracy. Similarly, we plotted ROC curves to further evaluate the comprehensive performance of different model classifications, and MCNN performed best in both sets of experiments. Therefore, comparing the evaluation indicators of the two groups of experiments, the overall performance of the MCNN model was better than other models. The different results of the two sets of experiments may be related to the structure and characteristics of the model. Simple structured networks such as MCNN and AlexNet can learn useful information better, while the complex network structure of ResNet and SqueezeNet does not work well instead. It also shows that complex deep learning networks are not always suitable for the feature mining of small samples. It is not necessary that the more the network structure is complex, the more effective the model is.

Previous research has indicated that the AgNPs/PSB composite substrate possesses favorable stability. By establishing a PCA-SVM model, the authors have demonstrated the superiority of combining SERS technology with machine learning algorithms for disease screening purposes. This study shows the feasibility of SERS technology based on annealed AgNPs/PSB composite substrates combined with deep learning algorithms for the diagnosis of immune diseases and chronic kidney disease, which has a great development prospect and research value in the early identification and screening of immune diseases and chronic kidney disease. However, there may be limitations in the study because of the limited sample size of the current study. Thus, we intend to collect more sample data in the future to evaluate further the effectiveness of SERS technology combined with deep learning for the screening of immune diseases and chronic kidney disease. Through the validation analysis of this exploratory study, SERS technology combined with strong deep learning algorithms can be innovatively extended to the research of different diseases and the screening of special populations.

## Conclusion

In this study, we developed an annealed AgNPs/PSB composite SERS substrate using PSB as a substrate, synthesized by an immersion plating and heat treatment method, and used serum SERS spectroscopy combined with deep learning algorithms achieved rapid and accurate diagnosis of patients with different diseases. For HC/pSS and HC/DN, we constructed two sets of experiments, established five deep learning classification models, and used five-fold cross-validation to ensure the experiments' reliability further. The results show that the MCNN algorithm is the most stable, with high accuracy, sensitivity and precision. Therefore, this study shows that SERS technology based on annealed AgNPs/PSB composite substrate combined with deep learning algorithm has a greater developmental prospect and research value in the early identification and screening of immune diseases and chronic kidney disease, providing reference ideas for non-invasive and rapid clinical medical diagnosis of patients.

### Supplementary Information

Supplementary Information.

## Data Availability

The datasets generated and analysed during the current study are not publicly available due to the nature of this research but are available from the corresponding author on reasonable request.
