# Supplementary material for: Application of serum SERS technology combined with deep learning algorithm in the rapid diagnosis of immune diseases and chronic kidney disease
Source: Sci Rep. 2023 Sep 21;13:15719. doi: 10.1038/s41598-023-42719-5 (PMC10514316; doi:10.1038/s41598-023-42719-5)
Supplement: Supplementary file 1 — Supplementary Information. [file 41598_2023_42719_MOESM1_ESM.docx]

**Table S1.** Five-fold CV results for AlexNet model in HC vs pSS

| **Number of CV** | **Accuracy** | **Sensitivity** | **Specificity** | **Precision** |
| --- | --- | --- | --- | --- |
| One | 86.7% | 100% | 66.7% | 81.8% |
| Two | 86.7% | 100% | 66.7% | 81.8% |
| Three | 93.3% | 100% | 83.3% | 90.0% |
| Four | 86.7% | 100% | 66.7% | 81.8% |
| Five | 93.3% | 100% | 83.3% | 90.0% |

**Table S2.** The predicted value of the Five-fold CV results of the AlexNet model in HC vs pSS

| Number of spectral data | Predicted value | | Predicted value | | Predicted value | | Predicted value | | Predicted value | |
| --- | --- | --- | --- | --- | --- | --- | --- | --- | --- | --- |
|  | One-fold CV | | Two-fold CV | | Three-fold CV | | Four-fold CV | | Five-fold CV | |
|  | HC | pSS | HC | pSS | HC | pSS | HC | pSS | HC | pSS |
| 1 | 3.68E-01 | 6.32E-01 | 1.16E-01 | 8.84E-01 | 7.30E-01 | 2.70E-01 | 8.64E-01 | 1.36E-01 | 6.43E-01 | 3.57E-01 |
| 2 | 1.29E-07 | 1.00E+00 | 6.20E-12 | 1.00E+00 | 3.17E-09 | 1.00E+00 | 1.48E-10 | 1.00E+00 | 9.78E-13 | 1.00E+00 |
| 3 | 8.20E-01 | 1.80E-01 | 9.17E-01 | 8.32E-02 | 9.56E-01 | 4.43E-02 | 9.62E-01 | 3.75E-02 | 9.91E-01 | 8.98E-03 |
| 4 | 1.00E+00 | 4.76E-15 | 1.00E+00 | 1.63E-20 | 1.00E+00 | 9.94E-18 | 1.00E+00 | 1.07E-18 | 1.00E+00 | 3.20E-22 |
| 5 | 1.00E+00 | 1.11E-20 | 1.00E+00 | 8.53E-30 | 1.00E+00 | 1.08E-25 | 1.00E+00 | 2.21E-28 | 1.00E+00 | 7.95E-33 |
| 6 | 9.97E-01 | 3.20E-03 | 9.89E-01 | 1.10E-02 | 9.51E-01 | 4.86E-02 | 3.93E-01 | 6.07E-01 | 9.66E-01 | 3.41E-02 |
| 7 | 1.08E-21 | 1.00E+00 | 2.55E-33 | 1.00E+00 | 9.56E-27 | 1.00E+00 | 3.26E-31 | 1.00E+00 | 3.74E-35 | 1.00E+00 |
| 8 | 5.22E-18 | 1.00E+00 | 3.26E-28 | 1.00E+00 | 1.36E-25 | 1.00E+00 | 7.55E-29 | 1.00E+00 | 1.67E-32 | 1.00E+00 |
| 9 | 1.08E-21 | 1.00E+00 | 2.55E-33 | 1.00E+00 | 9.56E-27 | 1.00E+00 | 3.26E-31 | 1.00E+00 | 3.74E-35 | 1.00E+00 |
| 10 | 4.66E-12 | 1.00E+00 | 3.33E-18 | 1.00E+00 | 5.32E-11 | 1.00E+00 | 4.37E-14 | 1.00E+00 | 7.84E-16 | 1.00E+00 |
| 11 | 1.41E-05 | 1.00E+00 | 5.04E-09 | 1.00E+00 | 4.07E-06 | 1.00E+00 | 1.05E-07 | 1.00E+00 | 8.29E-09 | 1.00E+00 |
| 12 | 5.67E-19 | 1.00E+00 | 2.95E-30 | 1.00E+00 | 1.72E-26 | 1.00E+00 | 1.25E-30 | 1.00E+00 | 2.78E-34 | 1.00E+00 |
| 13 | 1.03E-28 | 1.00E+00 | 0.00E+00 | 1.00E+00 | 1.98E-35 | 1.00E+00 | 0.00E+00 | 1.00E+00 | 0.00E+00 | 1.00E+00 |
| 14 | 1.92E-22 | 1.00E+00 | 8.40E-35 | 1.00E+00 | 1.55E-29 | 1.00E+00 | 4.24E-33 | 1.00E+00 | 6.69E-37 | 1.00E+00 |
| 15 | 4.01E-23 | 1.00E+00 | 1.07E-35 | 1.00E+00 | 7.02E-31 | 1.00E+00 | 7.27E-35 | 1.00E+00 | 0.00E+00 | 1.00E+00 |

The vertical axis of Supplementary Figures S1~S50 below represents the predicted value of the model for each test spectral data. For example, the label of HC is 0, and the closer the predicted value of HC is to 0, the more accurate it is; the label of pSS is 1, and the label of pSS The closer the predicted value is to 1, the more accurate it is. The abscissa indicates the number of the spectral data.





**Figure S1.** Stripplot of One-fold CV classification results of AlexNet model in HC vs pSS





**Figure S2.** Stripplot of Two-fold CV classification results of AlexNet model in HC vs pSS





**Figure S3.** Stripplot of Three-fold CV classification results of AlexNet model in HC vs pSS





**Figure S4.** Stripplot of Four-fold CV classification results of AlexNet model in HC vs pSS





**Figure S5.** Stripplot of Five-fold CV classification results of AlexNet model in HC vs pSS

**Table S3.** Five-fold CV results for ResNet model in HC vs pSS

| **Number of CV** | **Accuracy** | **Sensitivity** | **Specificity** | **Precision** |
| --- | --- | --- | --- | --- |
| One | 86.7% | 100% | 66.7% | 81.8% |
| Two | 80.0% | 100% | 50.0% | 75.0% |
| Three | 80.0% | 100% | 50.0% | 75.0% |
| Four | 86.7% | 100% | 66.7% | 81.8% |
| Five | 80.0% | 88.9% | 66.7% | 80.0% |

**Table S4.** The predicted value of the Five-fold CV results of the ResNet model in HC vs pSS

| Number of spectral data | Predicted value | | Predicted value | | Predicted value | | Predicted value | | Predicted value | |
| --- | --- | --- | --- | --- | --- | --- | --- | --- | --- | --- |
|  | One-fold CV | | Two-fold CV | | Three-fold CV | | Four-fold CV | | Five-fold CV | |
|  | HC | pSS | HC | pSS | HC | pSS | HC | pSS | HC | pSS |
| 1 | 8.07E-01 | 1.93E-01 | 9.98E-01 | 1.56E-03 | 1.00E+00 | 1.79E-06 | 1.00E+00 | 4.11E-08 | 1.00E+00 | 1.11E-08 |
| 2 | 2.41E-18 | 1.00E+00 | 1.62E-17 | 1.00E+00 | 3.21E-16 | 1.00E+00 | 2.50E-16 | 1.00E+00 | 1.73E-16 | 1.00E+00 |
| 3 | 5.35E-01 | 4.65E-01 | 4.98E-01 | 5.02E-01 | 4.80E-01 | 5.20E-01 | 1.00E+00 | 6.51E-06 | 1.00E+00 | 3.38E-05 |
| 4 | 1.00E+00 | 2.26E-21 | 1.00E+00 | 1.14E-21 | 1.00E+00 | 7.01E-21 | 1.00E+00 | 1.60E-20 | 1.00E+00 | 4.72E-21 |
| 5 | 1.00E+00 | 1.14E-28 | 1.00E+00 | 7.87E-29 | 1.00E+00 | 5.57E-28 | 1.00E+00 | 7.38E-28 | 1.00E+00 | 7.49E-28 |
| 6 | 4.14E-04 | 1.00E+00 | 1.38E-03 | 9.99E-01 | 1.26E-03 | 9.99E-01 | 2.21E-03 | 9.98E-01 | 5.79E-04 | 9.99E-01 |
| 7 | 0.00E+00 | 1.00E+00 | 0.00E+00 | 1.00E+00 | 0.00E+00 | 1.00E+00 | 0.00E+00 | 1.00E+00 | 0.00E+00 | 1.00E+00 |
| 8 | 0.00E+00 | 1.00E+00 | 0.00E+00 | 1.00E+00 | 0.00E+00 | 1.00E+00 | 0.00E+00 | 1.00E+00 | 0.00E+00 | 1.00E+00 |
| 9 | 0.00E+00 | 1.00E+00 | 0.00E+00 | 1.00E+00 | 0.00E+00 | 1.00E+00 | 0.00E+00 | 1.00E+00 | 0.00E+00 | 1.00E+00 |
| 10 | 1.42E-29 | 1.00E+00 | 2.14E-27 | 1.00E+00 | 1.04E-25 | 1.00E+00 | 6.82E-17 | 1.00E+00 | 1.12E-16 | 1.00E+00 |
| 11 | 2.62E-07 | 1.00E+00 | 4.59E-05 | 1.00E+00 | 4.66E-06 | 1.00E+00 | 1.40E-01 | 8.60E-01 | 8.18E-01 | 1.82E-01 |
| 12 | 0.00E+00 | 1.00E+00 | 0.00E+00 | 1.00E+00 | 0.00E+00 | 1.00E+00 | 0.00E+00 | 1.00E+00 | 7.12E-38 | 1.00E+00 |
| 13 | 0.00E+00 | 1.00E+00 | 0.00E+00 | 1.00E+00 | 0.00E+00 | 1.00E+00 | 0.00E+00 | 1.00E+00 | 0.00E+00 | 1.00E+00 |
| 14 | 0.00E+00 | 1.00E+00 | 3.97E-37 | 1.00E+00 | 8.23E-34 | 1.00E+00 | 1.62E-28 | 1.00E+00 | 1.99E-27 | 1.00E+00 |
| 15 | 0.00E+00 | 1.00E+00 | 0.00E+00 | 1.00E+00 | 0.00E+00 | 1.00E+00 | 0.00E+00 | 1.00E+00 | 0.00E+00 | 1.00E+00 |

**

**

**Figure S6.** Stripplot of One-fold CV classification results of ResNet model in HC vs pSS





**Figure S7.** Stripplot of Two-fold CV classification results of ResNet model in HC vs pSS





**Figure S8.** Stripplot of Three-fold CV classification results of ResNet model in HC vs pSS





**Figure S9.** Stripplot of Four-fold CV classification results of ResNet model in HC vs pSS

**

**

**Figure S10.** Stripplot of Five-fold CV classification results of ResNet model in HC vs pSS

**Table S5.** Five-fold CV results for SqueezeNet model in HC vs pSS

| **Number of CV** | **Accuracy** | **Sensitivity** | **Specificity** | **Precision** |
| --- | --- | --- | --- | --- |
| One | 93.3% | 100% | 83.3% | 90.0% |
| Two | 93.3% | 100% | 83.3% | 90.0% |
| Three | 93.3% | 100% | 83.3% | 90.0% |
| Four | 86.7% | 88.9% | 83.3% | 88.9% |
| Five | 93.3% | 100% | 83.3% | 90.0% |

**Table S6.** The predicted value of the Five-fold CV results of the SqueezeNet model

in HC vs pSS

| Number of spectral data | Predicted value | | Predicted value | | Predicted value | | Predicted value | | Predicted value | |
| --- | --- | --- | --- | --- | --- | --- | --- | --- | --- | --- |
|  | One-fold CV | | Two-fold CV | | Three-fold CV | | Four-fold CV | | Five-fold CV | |
|  | HC | pSS | HC | pSS | HC | pSS | HC | pSS | HC | pSS |
| 1 | 1.00E+00 | 6.44E-05 | 1.00E+00 | 3.67E-05 | 1.00E+00 | 7.03E-07 | 1.00E+00 | 8.11E-07 | 9.99E-01 | 8.89E-04 |
| 2 | 5.93E-03 | 9.94E-01 | 3.98E-03 | 9.96E-01 | 2.58E-02 | 9.74E-01 | 3.37E-02 | 9.66E-01 | 3.08E-04 | 1.00E+00 |
| 3 | 9.87E-01 | 1.35E-02 | 9.87E-01 | 1.29E-02 | 1.00E+00 | 1.75E-04 | 1.00E+00 | 2.32E-04 | 9.82E-01 | 1.79E-02 |
| 4 | 1.00E+00 | 2.82E-04 | 1.00E+00 | 3.17E-05 | 1.00E+00 | 3.12E-06 | 1.00E+00 | 2.43E-06 | 1.00E+00 | 6.53E-05 |
| 5 | 9.98E-01 | 1.64E-03 | 1.00E+00 | 4.71E-05 | 1.00E+00 | 3.92E-06 | 1.00E+00 | 2.76E-06 | 9.99E-01 | 9.71E-04 |
| 6 | 9.97E-01 | 2.64E-03 | 9.98E-01 | 1.78E-03 | 1.00E+00 | 6.50E-05 | 1.00E+00 | 1.12E-04 | 9.98E-01 | 1.61E-03 |
| 7 | 4.70E-05 | 1.00E+00 | 1.20E-03 | 9.99E-01 | 1.23E-03 | 9.99E-01 | 1.09E-02 | 9.89E-01 | 1.25E-06 | 1.00E+00 |
| 8 | 8.12E-03 | 9.92E-01 | 1.07E-01 | 8.93E-01 | 2.45E-01 | 7.55E-01 | 6.73E-01 | 3.27E-01 | 4.46E-04 | 1.00E+00 |
| 9 | 4.70E-05 | 1.00E+00 | 1.20E-03 | 9.99E-01 | 1.23E-03 | 9.99E-01 | 1.09E-02 | 9.89E-01 | 1.25E-06 | 1.00E+00 |
| 10 | 4.11E-06 | 1.00E+00 | 4.66E-05 | 1.00E+00 | 2.21E-05 | 1.00E+00 | 9.65E-05 | 1.00E+00 | 7.97E-07 | 1.00E+00 |
| 11 | 5.51E-05 | 1.00E+00 | 2.66E-03 | 9.97E-01 | 3.76E-03 | 9.96E-01 | 2.25E-02 | 9.78E-01 | 1.40E-05 | 1.00E+00 |
| 12 | 1.36E-06 | 1.00E+00 | 3.82E-05 | 1.00E+00 | 2.93E-05 | 1.00E+00 | 1.79E-04 | 1.00E+00 | 2.44E-07 | 1.00E+00 |
| 13 | 4.77E-04 | 1.00E+00 | 6.36E-03 | 9.94E-01 | 4.01E-03 | 9.96E-01 | 3.05E-02 | 9.69E-01 | 4.95E-05 | 1.00E+00 |
| 14 | 1.05E-03 | 9.99E-01 | 1.00E-02 | 9.90E-01 | 1.50E-02 | 9.85E-01 | 3.38E-02 | 9.66E-01 | 2.03E-03 | 9.98E-01 |
| 15 | 1.93E-02 | 9.81E-01 | 6.10E-02 | 9.39E-01 | 1.77E-01 | 8.23E-01 | 3.34E-01 | 6.66E-01 | 4.54E-03 | 9.95E-01 |

**

**

**Figure S11.** Stripplot of One-fold CV classification results of SqueezeNet model in HC vs pSS

**

**

**Figure S12.** Stripplot of Two-fold CV classification results of SqueezeNet model in HC vs pSS





**Figure S13.** Stripplot of Three-fold CV classification results of SqueezeNet model in HC vs pSS





**Figure S14.** Stripplot of Four-fold CV classification results of SqueezeNet model in HC vs pSS





**Figure S15.** Stripplot of Five-fold CV classification results of SqueezeNet model in HC vs pSS

**Table S7.** Five-fold CV results for TCN model in HC vs pSS

| **Number of CV** | **Accuracy** | **Sensitivity** | **Specificity** | **Precision** |
| --- | --- | --- | --- | --- |
| One | 93.3% | 100% | 83.3% | 90.0% |
| Two | 93.3% | 100% | 83.3% | 90.0% |
| Three | 93.3% | 100% | 83.3% | 90.0% |
| Four | 93.3% | 100% | 83.3% | 90.0% |
| Five | 93.3% | 100% | 83.3% | 90.0% |

**Table S8.** The predicted value of the Five-fold CV results of the TCN model in HC vs pSS

| Number of spectral data | Predicted value | | Predicted value | | Predicted value | | Predicted value | | Predicted value | |
| --- | --- | --- | --- | --- | --- | --- | --- | --- | --- | --- |
|  | One-fold CV | | Two-fold CV | | Three-fold CV | | Four-fold CV | | Five-fold CV | |
|  | HC | pSS | HC | pSS | HC | pSS | HC | pSS | HC | pSS |
| 1 | 8.55E-01 | 1.45E-01 | 5.80E-01 | 4.20E-01 | 8.90E-01 | 1.10E-01 | 8.86E-01 | 1.14E-01 | 9.81E-01 | 1.88E-02 |
| 2 | 1.51E-01 | 8.49E-01 | 2.83E-01 | 7.17E-01 | 3.02E-01 | 6.98E-01 | 1.40E-01 | 8.60E-01 | 2.76E-01 | 7.24E-01 |
| 3 | 6.22E-01 | 3.78E-01 | 6.30E-01 | 3.70E-01 | 9.45E-01 | 5.53E-02 | 7.97E-01 | 2.03E-01 | 9.55E-01 | 4.46E-02 |
| 4 | 1.00E+00 | 4.18E-05 | 1.00E+00 | 1.16E-04 | 1.00E+00 | 3.42E-05 | 1.00E+00 | 1.48E-08 | 1.00E+00 | 7.74E-09 |
| 5 | 1.00E+00 | 2.44E-06 | 1.00E+00 | 4.79E-06 | 1.00E+00 | 5.47E-07 | 1.00E+00 | 4.34E-12 | 1.00E+00 | 9.25E-13 |
| 6 | 1.00E+00 | 4.94E-04 | 9.97E-01 | 3.45E-03 | 9.99E-01 | 5.17E-04 | 1.00E+00 | 1.79E-05 | 1.00E+00 | 5.72E-06 |
| 7 | 4.57E-04 | 1.00E+00 | 6.77E-04 | 9.99E-01 | 2.77E-06 | 1.00E+00 | 5.00E-07 | 1.00E+00 | 1.73E-08 | 1.00E+00 |
| 8 | 7.24E-03 | 9.93E-01 | 4.52E-03 | 9.95E-01 | 4.06E-04 | 1.00E+00 | 2.84E-05 | 1.00E+00 | 1.32E-05 | 1.00E+00 |
| 9 | 4.57E-04 | 1.00E+00 | 6.77E-04 | 9.99E-01 | 2.77E-06 | 1.00E+00 | 5.00E-07 | 1.00E+00 | 1.73E-08 | 1.00E+00 |
| 10 | 8.08E-05 | 1.00E+00 | 3.34E-04 | 1.00E+00 | 2.55E-06 | 1.00E+00 | 9.72E-07 | 1.00E+00 | 1.39E-08 | 1.00E+00 |
| 11 | 1.52E-03 | 9.98E-01 | 3.03E-03 | 9.97E-01 | 9.04E-05 | 1.00E+00 | 2.06E-05 | 1.00E+00 | 8.66E-07 | 1.00E+00 |
| 12 | 6.37E-06 | 1.00E+00 | 1.51E-05 | 1.00E+00 | 2.31E-07 | 1.00E+00 | 7.28E-10 | 1.00E+00 | 2.03E-11 | 1.00E+00 |
| 13 | 1.60E-02 | 9.84E-01 | 3.06E-02 | 9.69E-01 | 1.04E-04 | 1.00E+00 | 9.62E-04 | 9.99E-01 | 7.17E-05 | 1.00E+00 |
| 14 | 2.56E-02 | 9.74E-01 | 3.92E-02 | 9.61E-01 | 4.99E-04 | 1.00E+00 | 5.81E-04 | 9.99E-01 | 1.95E-04 | 1.00E+00 |
| 15 | 1.13E-02 | 9.89E-01 | 1.49E-02 | 9.85E-01 | 2.42E-04 | 1.00E+00 | 6.01E-05 | 1.00E+00 | 2.71E-05 | 1.00E+00 |





**Figure S16.** Stripplot of One-fold CV classification results of TCN model in HC vs pSS





**Figure S17.** Stripplot of Two-fold CV classification results of TCN model in HC vs pSS





**Figure S18.** Stripplot of Three-fold CV classification results of TCN model in HC vs pSS





**Figure S19.** Stripplot of Four-fold CV classification results of TCN model in HC vs pSS





**Figure S20.** Stripplot of Five-fold CV classification results of TCN model in HC vs pSS

**Table S9.** Five-fold CV results for MCNN model in HC vs pSS

| **Number of CV** | **Accuracy** | **Sensitivity** | **Specificity** | **Precision** |
| --- | --- | --- | --- | --- |
| One | 93.3% | 100% | 83.3% | 90.0% |
| Two | 93.3% | 100% | 83.3% | 90.0% |
| Three | 93.3% | 100% | 83.3% | 90.0% |
| Four | 93.3% | 100% | 83.3% | 90.0% |
| Five | 100% | 100% | 100% | 100% |

**Table S10.** The predicted value of the Five-fold CV results of the MCNN model in HC vs pSS

| Number of spectral data | Predicted value | | Predicted value | | Predicted value | | Predicted value | | Predicted value | |
| --- | --- | --- | --- | --- | --- | --- | --- | --- | --- | --- |
|  | One-fold CV | | Two-fold CV | | Three-fold CV | | Four-fold CV | | Five-fold CV | |
|  | HC | pSS | HC | pSS | HC | pSS | HC | pSS | HC | pSS |
| 1 | 5.43E-01 | 4.57E-01 | 9.72E-01 | 2.83E-02 | 5.30E-01 | 4.70E-01 | 9.99E-01 | 8.27E-04 | 9.99E-01 | 1.32E-03 |
| 2 | 1.26E-02 | 9.87E-01 | 1.46E-01 | 8.54E-01 | 3.19E-04 | 1.00E+00 | 4.15E-01 | 5.85E-01 | 9.15E-01 | 8.50E-02 |
| 3 | 6.45E-01 | 3.55E-01 | 9.55E-01 | 4.49E-02 | 5.17E-01 | 4.83E-01 | 9.98E-01 | 2.46E-03 | 9.85E-01 | 1.54E-02 |
| 4 | 1.00E+00 | 1.46E-06 | 1.00E+00 | 1.56E-07 | 1.00E+00 | 1.79E-09 | 1.00E+00 | 8.40E-15 | 1.00E+00 | 2.51E-10 |
| 5 | 1.00E+00 | 3.28E-08 | 1.00E+00 | 4.95E-10 | 1.00E+00 | 3.31E-12 | 1.00E+00 | 3.17E-19 | 1.00E+00 | 3.96E-14 |
| 6 | 1.00E+00 | 3.74E-04 | 9.98E-01 | 1.57E-03 | 1.00E+00 | 4.80E-05 | 1.00E+00 | 1.65E-07 | 1.00E+00 | 1.97E-04 |
| 7 | 7.15E-06 | 1.00E+00 | 5.09E-08 | 1.00E+00 | 1.19E-10 | 1.00E+00 | 3.55E-07 | 1.00E+00 | 4.91E-07 | 1.00E+00 |
| 8 | 3.68E-04 | 1.00E+00 | 1.46E-04 | 1.00E+00 | 5.01E-07 | 1.00E+00 | 5.58E-04 | 9.99E-01 | 2.77E-02 | 9.72E-01 |
| 9 | 7.15E-06 | 1.00E+00 | 5.09E-08 | 1.00E+00 | 1.19E-10 | 1.00E+00 | 3.55E-07 | 1.00E+00 | 4.91E-07 | 1.00E+00 |
| 10 | 2.17E-04 | 1.00E+00 | 5.73E-06 | 1.00E+00 | 9.72E-08 | 1.00E+00 | 1.86E-02 | 9.81E-01 | 2.18E-05 | 1.00E+00 |
| 11 | 2.34E-03 | 9.98E-01 | 3.83E-04 | 1.00E+00 | 7.58E-06 | 1.00E+00 | 4.34E-02 | 9.57E-01 | 3.56E-03 | 9.96E-01 |
| 12 | 2.29E-07 | 1.00E+00 | 6.05E-10 | 1.00E+00 | 3.86E-13 | 1.00E+00 | 4.04E-11 | 1.00E+00 | 1.22E-09 | 1.00E+00 |
| 13 | 7.22E-06 | 1.00E+00 | 1.07E-06 | 1.00E+00 | 2.24E-09 | 1.00E+00 | 6.35E-05 | 1.00E+00 | 3.23E-04 | 1.00E+00 |
| 14 | 3.32E-05 | 1.00E+00 | 6.74E-05 | 1.00E+00 | 1.05E-07 | 1.00E+00 | 9.89E-04 | 9.99E-01 | 6.12E-02 | 9.39E-01 |
| 15 | 3.36E-05 | 1.00E+00 | 7.22E-05 | 1.00E+00 | 1.16E-07 | 1.00E+00 | 3.14E-04 | 1.00E+00 | 2.98E-02 | 9.70E-01 |

**

**

**Figure S21.** Stripplot of One-fold CV classification results of MCNN model in HC vs pSS





**Figure S22.** Stripplot of Two-fold CV classification results of MCNN model in HC vs pSS





**Figure S23.** Stripplot of Three-fold CV classification results of MCNN model in HC vs pSS





**Figure S24.** Stripplot of Four-fold CV classification results of MCNN model in HC vs pSS





**Figure S25.** Stripplot of Five-fold CV classification results of MCNN model in HC vs pSS

**Table S11.** Five-fold CV results for AlexNet model in HC vs DN

| **Number of CV** | **Accuracy** | **Sensitivity** | **Specificity** | **Precision** |
| --- | --- | --- | --- | --- |
| One | 80.0% | 88.9% | 66.7% | 80.0% |
| Two | 86.7% | 88.9% | 83.3% | 88.9% |
| Three | 86.7% | 100% | 66.7% | 81.8% |
| Four | 86.7% | 100% | 66.7% | 81.8% |
| Five | 100% | 100% | 100% | 100% |

**Table S12.** The predicted value of the Five-fold CV results of the AlexNet model

in HC vs DN

| Number of spectral data | Predicted value | | Predicted value | | Predicted value | | Predicted value | | Predicted value | |
| --- | --- | --- | --- | --- | --- | --- | --- | --- | --- | --- |
|  | One-fold CV | | Two-fold CV | | Three-fold CV | | Four-fold CV | | Five-fold CV | |
|  | HC | DN | HC | DN | HC | DN | HC | DN | HC | DN |
| 1 | 5.64E-03 | 9.94E-01 | 9.21E-01 | 7.87E-02 | 3.92E-02 | 9.61E-01 | 5.84E-04 | 9.99E-01 | 1.00E+00 | 4.40E-08 |
| 2 | 6.10E-04 | 9.99E-01 | 4.87E-09 | 1.00E+00 | 8.86E-11 | 1.00E+00 | 2.32E-07 | 1.00E+00 | 1.00E+00 | 1.11E-16 |
| 3 | 1.00E+00 | 1.42E-04 | 1.00E+00 | 1.74E-08 | 1.00E+00 | 1.12E-08 | 1.00E+00 | 5.25E-15 | 1.00E+00 | 5.12E-10 |
| 4 | 1.00E+00 | 6.58E-24 | 1.00E+00 | 0.00E+00 | 1.00E+00 | 0.00E+00 | 1.00E+00 | 0.00E+00 | 1.00E+00 | 0.00E+00 |
| 5 | 1.00E+00 | 2.94E-30 | 1.00E+00 | 0.00E+00 | 1.00E+00 | 0.00E+00 | 1.00E+00 | 0.00E+00 | 1.00E+00 | 0.00E+00 |
| 6 | 1.00E+00 | 1.83E-08 | 1.00E+00 | 2.12E-19 | 1.00E+00 | 1.71E-11 | 1.00E+00 | 9.81E-24 | 1.00E+00 | 3.75E-14 |
| 7 | 4.30E-01 | 5.70E-01 | 3.51E-02 | 9.65E-01 | 2.44E-37 | 1.00E+00 | 5.53E-24 | 1.00E+00 | 0.00E+00 | 1.00E+00 |
| 8 | 1.00E+00 | 1.28E-06 | 1.00E+00 | 9.07E-09 | 2.33E-28 | 1.00E+00 | 2.01E-14 | 1.00E+00 | 7.43E-37 | 1.00E+00 |
| 9 | 0.00E+00 | 1.00E+00 | 0.00E+00 | 1.00E+00 | 0.00E+00 | 1.00E+00 | 0.00E+00 | 1.00E+00 | 0.00E+00 | 1.00E+00 |
| 10 | 4.72E-21 | 1.00E+00 | 0.00E+00 | 1.00E+00 | 0.00E+00 | 1.00E+00 | 7.50E-37 | 1.00E+00 | 2.57E-34 | 1.00E+00 |
| 11 | 2.57E-37 | 1.00E+00 | 0.00E+00 | 1.00E+00 | 0.00E+00 | 1.00E+00 | 0.00E+00 | 1.00E+00 | 0.00E+00 | 1.00E+00 |
| 12 | 6.94E-37 | 1.00E+00 | 0.00E+00 | 1.00E+00 | 0.00E+00 | 1.00E+00 | 0.00E+00 | 1.00E+00 | 0.00E+00 | 1.00E+00 |
| 13 | 3.97E-10 | 1.00E+00 | 7.31E-33 | 1.00E+00 | 7.46E-38 | 1.00E+00 | 4.16E-35 | 1.00E+00 | 5.72E-35 | 1.00E+00 |
| 14 | 9.41E-29 | 1.00E+00 | 0.00E+00 | 1.00E+00 | 0.00E+00 | 1.00E+00 | 0.00E+00 | 1.00E+00 | 0.00E+00 | 1.00E+00 |
| 15 | 1.19E-24 | 1.00E+00 | 0.00E+00 | 1.00E+00 | 0.00E+00 | 1.00E+00 | 0.00E+00 | 1.00E+00 | 0.00E+00 | 1.00E+00 |





**Figure S26.** Stripplot of One-fold CV classification results of AlexNet model in HC vs DN





**Figure S27.** Stripplot of Two-fold CV classification results of AlexNet model in HC vs DN





**Figure S28.** Stripplot of Three-fold CV classification results of AlexNet model in HC vs DN





**Figure S29.** Stripplot of Four-fold CV classification results of AlexNet model in HC vs DN





**Figure S30.** Stripplot of Five-fold CV classification results of AlexNet model in HC vs DN

**Table S13.** Five-fold CV results for ResNet model in HC vs DN

| **Number of CV** | **Accuracy** | **Sensitivity** | **Specificity** | **Precision** |
| --- | --- | --- | --- | --- |
| One | 93.3% | 88.9% | 100% | 100% |
| Two | 93.3% | 100% | 83.3% | 90.0% |
| Three | 66.7% | 88.9% | 33.3% | 66.7% |
| Four | 73.3% | 77.8% | 66.7% | 77.8% |
| Five | 73.3% | 66.7% | 83.3% | 85.7% |

**Table S14.** The predicted value of the Five-fold CV results of the ResNet model in HC vs DN

| Number of spectral data | Predicted value | | Predicted value | | Predicted value | | Predicted value | | Predicted value | |
| --- | --- | --- | --- | --- | --- | --- | --- | --- | --- | --- |
|  | One-fold CV | | Two-fold CV | | Three-fold CV | | Four-fold CV | | Five-fold CV | |
|  | HC | DN | HC | DN | HC | DN | HC | DN | HC | DN |
| 1 | 1.00E+00 | 1.37E-05 | 1.00E+00 | 1.96E-13 | 1.71E-01 | 8.29E-01 | 1.25E-21 | 1.00E+00 | 1.00E+00 | 3.23E-13 |
| 2 | 1.00E+00 | 1.62E-05 | 6.90E-33 | 1.00E+00 | 3.42E-08 | 1.00E+00 | 0.00E+00 | 1.00E+00 | 0.00E+00 | 1.00E+00 |
| 3 | 1.00E+00 | 5.46E-08 | 1.00E+00 | 1.94E-23 | 7.84E-05 | 1.00E+00 | 1.00E+00 | 1.69E-37 | 1.00E+00 | 0.00E+00 |
| 4 | 1.00E+00 | 5.44E-21 | 1.00E+00 | 0.00E+00 | 1.00E+00 | 0.00E+00 | 1.00E+00 | 0.00E+00 | 1.00E+00 | 0.00E+00 |
| 5 | 1.00E+00 | 4.03E-25 | 1.00E+00 | 0.00E+00 | 1.00E+00 | 0.00E+00 | 1.00E+00 | 0.00E+00 | 1.00E+00 | 0.00E+00 |
| 6 | 1.00E+00 | 1.82E-09 | 1.00E+00 | 1.27E-19 | 4.40E-33 | 1.00E+00 | 1.00E+00 | 0.00E+00 | 1.00E+00 | 0.00E+00 |
| 7 | 4.06E-09 | 1.00E+00 | 1.25E-16 | 1.00E+00 | 9.33E-36 | 1.00E+00 | 3.26E-04 | 1.00E+00 | 7.34E-01 | 2.66E-01 |
| 8 | 9.47E-05 | 1.00E+00 | 3.40E-10 | 1.00E+00 | 2.58E-21 | 1.00E+00 | 1.00E+00 | 1.02E-09 | 1.00E+00 | 2.32E-17 |
| 9 | 1.00E+00 | 7.95E-05 | 1.38E-11 | 1.00E+00 | 1.00E+00 | 6.11E-23 | 1.00E+00 | 0.00E+00 | 1.00E+00 | 0.00E+00 |
| 10 | 1.30E-15 | 1.00E+00 | 0.00E+00 | 1.00E+00 | 0.00E+00 | 1.00E+00 | 0.00E+00 | 1.00E+00 | 0.00E+00 | 1.00E+00 |
| 11 | 4.80E-19 | 1.00E+00 | 0.00E+00 | 1.00E+00 | 0.00E+00 | 1.00E+00 | 0.00E+00 | 1.00E+00 | 0.00E+00 | 1.00E+00 |
| 12 | 1.90E-10 | 1.00E+00 | 0.00E+00 | 1.00E+00 | 0.00E+00 | 1.00E+00 | 0.00E+00 | 1.00E+00 | 0.00E+00 | 1.00E+00 |
| 13 | 4.89E-01 | 5.11E-01 | 0.00E+00 | 1.00E+00 | 4.07E-38 | 1.00E+00 | 0.00E+00 | 1.00E+00 | 0.00E+00 | 1.00E+00 |
| 14 | 3.18E-06 | 1.00E+00 | 0.00E+00 | 1.00E+00 | 7.11E-21 | 1.00E+00 | 0.00E+00 | 1.00E+00 | 0.00E+00 | 1.00E+00 |
| 15 | 4.15E-04 | 1.00E+00 | 0.00E+00 | 1.00E+00 | 7.03E-36 | 1.00E+00 | 0.00E+00 | 1.00E+00 | 0.00E+00 | 1.00E+00 |

**

**

**Figure S31.** Stripplot of One-fold CV classification results of ResNet model in HC vs DN





**Figure S32.** Stripplot of Two-fold CV classification results of ResNet model in HC vs DN





**Figure S33.** Stripplot of Three-fold CV classification results of ResNet model in HC vs DN





**Figure S34.** Stripplot of Four-fold CV classification results of ResNet model in HC vs DN





**Figure S35.** Stripplot of Five-fold CV classification results of ResNet model in HC vs DN

**Table S15.** Five-fold CV results for SqueezeNet model in HC vs DN

| **Number of CV** | **Accuracy** | **Sensitivity** | **Specificity** | **Precision** |
| --- | --- | --- | --- | --- |
| One | 73.3% | 55.6% | 100% | 100% |
| Two | 73.3% | 55.6% | 100% | 100% |
| Three | 73.3% | 55.6% | 100% | 100% |
| Four | 73.3% | 55.6% | 100% | 100% |
| Five | 86.7% | 100% | 66.7% | 81.8% |

**Table S16.** The predicted value of the Five-fold CV results of the SqueezeNet model

in HC vs DN

| Number of spectral data | Predicted value | | Predicted value | | Predicted value | | Predicted value | | Predicted value | |
| --- | --- | --- | --- | --- | --- | --- | --- | --- | --- | --- |
|  | One-fold CV | | Two-fold CV | | Three-fold CV | | Four-fold CV | | Five-fold CV | |
|  | HC | DN | HC | DN | HC | DN | HC | DN | HC | DN |
| 1 | 9.71E-01 | 2.90E-02 | 9.92E-01 | 8.32E-03 | 9.89E-01 | 1.11E-02 | 9.99E-01 | 7.32E-04 | 4.76E-02 | 9.52E-01 |
| 2 | 7.58E-01 | 2.42E-01 | 8.35E-01 | 1.65E-01 | 8.77E-01 | 1.23E-01 | 9.73E-01 | 2.72E-02 | 3.37E-09 | 1.00E+00 |
| 3 | 1.00E+00 | 5.73E-05 | 1.00E+00 | 4.33E-04 | 9.99E-01 | 5.34E-04 | 1.00E+00 | 7.59E-05 | 9.98E-01 | 1.70E-03 |
| 4 | 1.00E+00 | 8.98E-08 | 1.00E+00 | 3.26E-07 | 1.00E+00 | 6.36E-07 | 1.00E+00 | 1.22E-07 | 1.00E+00 | 5.85E-10 |
| 5 | 1.00E+00 | 1.59E-09 | 1.00E+00 | 2.10E-08 | 1.00E+00 | 5.06E-08 | 1.00E+00 | 2.14E-09 | 1.00E+00 | 2.13E-07 |
| 6 | 9.98E-01 | 2.47E-03 | 9.24E-01 | 7.58E-02 | 8.15E-01 | 1.85E-01 | 9.84E-01 | 1.61E-02 | 5.96E-01 | 4.04E-01 |
| 7 | 9.73E-01 | 2.65E-02 | 9.58E-01 | 4.17E-02 | 9.09E-01 | 9.11E-02 | 9.80E-01 | 2.05E-02 | 2.36E-07 | 1.00E+00 |
| 8 | 9.98E-01 | 1.79E-03 | 9.99E-01 | 8.10E-04 | 9.97E-01 | 2.65E-03 | 9.99E-01 | 6.08E-04 | 3.05E-04 | 1.00E+00 |
| 9 | 9.95E-01 | 4.59E-03 | 9.96E-01 | 3.76E-03 | 9.88E-01 | 1.15E-02 | 9.99E-01 | 1.40E-03 | 9.92E-04 | 9.99E-01 |
| 10 | 1.37E-04 | 1.00E+00 | 9.16E-06 | 1.00E+00 | 1.04E-05 | 1.00E+00 | 6.68E-05 | 1.00E+00 | 6.95E-19 | 1.00E+00 |
| 11 | 8.20E-08 | 1.00E+00 | 4.76E-09 | 1.00E+00 | 9.41E-09 | 1.00E+00 | 3.14E-08 | 1.00E+00 | 1.56E-21 | 1.00E+00 |
| 12 | 1.82E-07 | 1.00E+00 | 6.76E-08 | 1.00E+00 | 8.85E-08 | 1.00E+00 | 9.06E-07 | 1.00E+00 | 4.17E-21 | 1.00E+00 |
| 13 | 5.02E-03 | 9.95E-01 | 7.24E-03 | 9.93E-01 | 6.79E-03 | 9.93E-01 | 2.77E-02 | 9.72E-01 | 2.44E-17 | 1.00E+00 |
| 14 | 7.61E-03 | 9.92E-01 | 1.37E-03 | 9.99E-01 | 1.34E-03 | 9.99E-01 | 1.89E-02 | 9.81E-01 | 6.87E-18 | 1.00E+00 |
| 15 | 9.99E-01 | 8.35E-04 | 9.93E-01 | 6.53E-03 | 9.87E-01 | 1.28E-02 | 9.96E-01 | 3.60E-03 | 7.68E-12 | 1.00E+00 |





**Figure S36.** Stripplot of One-fold CV classification results of SqueezeNet model in HC vs DN





**Figure S37.** Stripplot of Two-fold CV classification results of SqueezeNet model in HC vs DN





**Figure S38.** Stripplot of Three-fold CV classification results of SqueezeNet model in HC vs DN





**Figure S39.** Stripplot of Four-fold CV classification results of SqueezeNet model in HC vs DN





**Figure S40.** Stripplot of Five-fold CV classification results of SqueezeNet model in HC vs DN

**Table S17.** Five-fold CV results for TCN model in HC vs DN

| **Number of CV** | **Accuracy** | **Sensitivity** | **Specificity** | **Precision** |
| --- | --- | --- | --- | --- |
| One | 80.0% | 100% | 50.0% | 75.0% |
| Two | 73.3% | 88.9% | 50.0% | 72.7% |
| Three | 80.0% | 100% | 50.0% | 75.0% |
| Four | 86.7% | 100% | 66.7% | 81.8% |
| Five | 93.3% | 100% | 83.3% | 90.0% |

**Table S18.** The predicted value of the Five-fold CV results of the TCN model in HC vs DN

| Number of spectral data | Predicted value | | Predicted value | | Predicted value | | Predicted value | | Predicted value | |
| --- | --- | --- | --- | --- | --- | --- | --- | --- | --- | --- |
|  | One-fold CV | | Two-fold CV | | Three-fold CV | | Four-fold CV | | Five-fold CV | |
|  | HC | DN | HC | DN | HC | DN | HC | DN | HC | DN |
| 1 | 2.45E-01 | 7.55E-01 | 5.54E-03 | 9.94E-01 | 1.99E-01 | 8.01E-01 | 9.59E-01 | 4.06E-02 | 9.05E-01 | 9.46E-02 |
| 2 | 4.28E-02 | 9.57E-01 | 2.03E-06 | 1.00E+00 | 3.14E-06 | 1.00E+00 | 2.30E-03 | 9.98E-01 | 6.01E-01 | 3.99E-01 |
| 3 | 6.15E-01 | 3.85E-01 | 1.81E-01 | 8.19E-01 | 9.23E-01 | 7.67E-02 | 9.73E-01 | 2.66E-02 | 6.12E-01 | 3.88E-01 |
| 4 | 9.77E-01 | 2.33E-02 | 1.00E+00 | 2.07E-04 | 1.00E+00 | 8.69E-06 | 9.99E-01 | 5.15E-04 | 5.71E-01 | 4.29E-01 |
| 5 | 9.99E-01 | 1.21E-03 | 1.00E+00 | 4.30E-06 | 1.00E+00 | 7.53E-09 | 1.00E+00 | 4.80E-08 | 8.96E-01 | 1.04E-01 |
| 6 | 6.70E-02 | 9.33E-01 | 5.69E-01 | 4.31E-01 | 8.56E-02 | 9.14E-01 | 1.03E-06 | 1.00E+00 | 1.71E-01 | 8.29E-01 |
| 7 | 5.48E-02 | 9.45E-01 | 7.47E-03 | 9.93E-01 | 3.75E-05 | 1.00E+00 | 2.10E-11 | 1.00E+00 | 4.51E-02 | 9.55E-01 |
| 8 | 4.48E-01 | 5.52E-01 | 6.65E-01 | 3.35E-01 | 9.94E-02 | 9.01E-01 | 3.75E-08 | 1.00E+00 | 1.60E-01 | 8.40E-01 |
| 9 | 4.36E-01 | 5.64E-01 | 2.96E-03 | 9.97E-01 | 2.17E-01 | 7.83E-01 | 1.74E-05 | 1.00E+00 | 1.62E-01 | 8.38E-01 |
| 10 | 2.48E-04 | 1.00E+00 | 8.91E-13 | 1.00E+00 | 1.70E-13 | 1.00E+00 | 2.19E-11 | 1.00E+00 | 1.22E-02 | 9.88E-01 |
| 11 | 1.41E-05 | 1.00E+00 | 8.11E-16 | 1.00E+00 | 7.72E-17 | 1.00E+00 | 9.33E-15 | 1.00E+00 | 5.36E-03 | 9.95E-01 |
| 12 | 1.31E-05 | 1.00E+00 | 1.24E-14 | 1.00E+00 | 3.86E-16 | 1.00E+00 | 1.11E-14 | 1.00E+00 | 4.90E-03 | 9.95E-01 |
| 13 | 4.48E-03 | 9.96E-01 | 3.79E-09 | 1.00E+00 | 1.25E-09 | 1.00E+00 | 3.68E-08 | 1.00E+00 | 1.27E-01 | 8.73E-01 |
| 14 | 1.21E-03 | 9.99E-01 | 5.22E-11 | 1.00E+00 | 1.04E-11 | 1.00E+00 | 1.38E-09 | 1.00E+00 | 1.52E-01 | 8.48E-01 |
| 15 | 2.14E-03 | 9.98E-01 | 2.52E-12 | 1.00E+00 | 8.66E-13 | 1.00E+00 | 5.94E-10 | 1.00E+00 | 2.56E-01 | 7.44E-01 |

**

**

**Figure S41.** Stripplot of One-fold CV classification results of TCN model in HC vs DN





**Figure S42.** Stripplot of Two-fold CV classification results of TCN model in HC vs DN





**Figure S43.** Stripplot of Three-fold CV classification results of TCN model in HC vs DN





**Figure S44.** Stripplot of Four-fold CV classification results of TCN model in HC vs DN





**Figure S45.** Stripplot of Five-fold CV classification results of TCN model in HC vs DN

**Table S19.** Five-fold CV results for MCNN model in HC vs DN

| **Number of CV** | **Accuracy** | **Sensitivity** | **Specificity** | **Precision** |
| --- | --- | --- | --- | --- |
| One | 93.3% | 100% | 83.3% | 90.0% |
| Two | 93.3% | 100% | 83.3% | 90.0% |
| Three | 86.7% | 88.9% | 83.3% | 88.9% |
| Four | 86.7% | 88.9% | 83.3% | 88.9% |
| Five | 100% | 100% | 100% | 100% |

**Table S20.** The predicted value of the Five-fold CV results of the MCNN model in HC vs DN

| Number of spectral data | Predicted value | | Predicted value | | Predicted value | | Predicted value | | Predicted value | |
| --- | --- | --- | --- | --- | --- | --- | --- | --- | --- | --- |
|  | One-fold CV | | Two-fold CV | | Three-fold CV | | Four-fold CV | | Five-fold CV | |
|  | HC | DN | HC | DN | HC | DN | HC | DN | HC | DN |
| 1 | 9.98E-01 | 1.64E-03 | 1.00E+00 | 9.27E-05 | 1.00E+00 | 5.53E-05 | 1.00E+00 | 3.16E-06 | 1.00E+00 | 1.67E-19 |
| 2 | 1.18E-03 | 9.99E-01 | 7.89E-04 | 9.99E-01 | 7.73E-03 | 9.92E-01 | 1.94E-04 | 1.00E+00 | 9.67E-01 | 3.33E-02 |
| 3 | 1.00E+00 | 8.07E-07 | 1.00E+00 | 1.03E-08 | 1.00E+00 | 2.06E-05 | 1.00E+00 | 4.61E-07 | 1.00E+00 | 3.87E-14 |
| 4 | 1.00E+00 | 1.11E-18 | 1.00E+00 | 2.36E-20 | 1.00E+00 | 3.62E-16 | 1.00E+00 | 3.01E-29 | 1.00E+00 | 0.00E+00 |
| 5 | 1.00E+00 | 2.68E-25 | 1.00E+00 | 5.70E-28 | 1.00E+00 | 2.12E-22 | 1.00E+00 | 0.00E+00 | 1.00E+00 | 0.00E+00 |
| 6 | 9.26E-01 | 7.43E-02 | 7.20E-01 | 2.80E-01 | 9.06E-01 | 9.39E-02 | 1.00E+00 | 8.29E-08 | 1.00E+00 | 3.47E-08 |
| 7 | 3.53E-04 | 1.00E+00 | 6.00E-06 | 1.00E+00 | 1.08E-01 | 8.92E-01 | 1.07E-01 | 8.93E-01 | 8.99E-09 | 1.00E+00 |
| 8 | 1.87E-01 | 8.13E-01 | 1.19E-02 | 9.88E-01 | 9.61E-01 | 3.94E-02 | 9.94E-01 | 5.75E-03 | 1.49E-02 | 9.85E-01 |
| 9 | 3.59E-04 | 1.00E+00 | 2.51E-05 | 1.00E+00 | 3.28E-09 | 1.00E+00 | 2.24E-09 | 1.00E+00 | 1.29E-12 | 1.00E+00 |
| 10 | 1.69E-16 | 1.00E+00 | 7.32E-20 | 1.00E+00 | 1.28E-17 | 1.00E+00 | 7.06E-27 | 1.00E+00 | 0.00E+00 | 1.00E+00 |
| 11 | 1.36E-20 | 1.00E+00 | 6.94E-25 | 1.00E+00 | 3.53E-22 | 1.00E+00 | 4.22E-36 | 1.00E+00 | 0.00E+00 | 1.00E+00 |
| 12 | 1.16E-19 | 1.00E+00 | 1.32E-23 | 1.00E+00 | 1.58E-20 | 1.00E+00 | 1.63E-31 | 1.00E+00 | 0.00E+00 | 1.00E+00 |
| 13 | 2.57E-11 | 1.00E+00 | 1.82E-13 | 1.00E+00 | 2.35E-09 | 1.00E+00 | 1.80E-14 | 1.00E+00 | 6.09E-24 | 1.00E+00 |
| 14 | 3.06E-14 | 1.00E+00 | 4.06E-17 | 1.00E+00 | 4.10E-13 | 1.00E+00 | 1.71E-18 | 1.00E+00 | 5.75E-17 | 1.00E+00 |
| 15 | 4.35E-20 | 1.00E+00 | 1.19E-23 | 1.00E+00 | 9.55E-19 | 1.00E+00 | 2.04E-27 | 1.00E+00 | 3.71E-34 | 1.00E+00 |





**Figure S46.** Stripplot of One-fold CV classification results of MCNN model in HC vs DN





**Figure S47.** Stripplot of Two-fold CV classification results of MCNN model in HC vs DN





**Figure S48.** Stripplot of Three-fold CV classification results of MCNN model in HC vs DN





**Figure S49.** Stripplot of Four-fold CV classification results of MCNN model in HC vs DN





**Figure S50.** Stripplot of Five-fold CV classification results of MCNN model in HC vs DN





**Figure S51.** All raw spectra of HC





**Figure S52.** All raw spectra of pSS





**Figure S53.** All raw spectra of DN
